# Visual Quality and Symptomatology Following Implantation of a Non-Diffractive Extended Depth-of-Focus Intraocular Lens

**DOI:** 10.3390/jcm14134460

**Published:** 2025-06-23

**Authors:** Antonio Cano-Ortiz, Álvaro Sánchez-Ventosa, Timoteo González-Cruces, Marta Villalba-González, Francisco Javier Aguilar-Salazar, Juan J. Prados-Carmona, Carlos Carpena-Torres, Gonzalo Carracedo, Alberto Villarrubia

**Affiliations:** 1 Department of Anterior Segment, Cornea and Refractive Surgery, Hospital Arruzafa, 14012 Cordoba, Spain; antoniocanoortiz@gmail.com (A.C.-O.); alvarosventosa@gmail.com (Á.S.-V.); marta.villalba7@gmail.com (M.V.-G.); franaguilar@hospitalarruzafa.com (F.J.A.-S.); 2 Department of Health and Biomedical Sciences, Universidad Loyola Andalucía, 41703 Sevilla, Spain; 3 Department of Ophthalmology, Reina Sofia University Hospital, 14004 Cordoba, Spain; 4 Ocupharm Research Group, Department of Optometry and Vision, University Complutense of Madrid, 28040 Madrid, Spain; ccarpena@ucm.es (C.C.-T.); jgcarrac@ucm.es (G.C.)

**Keywords:** extended range, EDOF, defocus curve, visual acuity, contrast sensitivity

## Abstract

**Background/Objectives**: This study aimed to evaluate the visual quality and symptomatology of a non-diffractive extended depth-of-focus (EDoF) intraocular lens (IOL), the Elon 877PEY (Medicontur, Zsámbék, Hungary), three months after implantation. **Methods**: A cross-sectional case series study was conducted, with measurements taken three months post-implantation of the Elon IOL. A total of 56 implanted eyes from 28 patients (mean age: 64.5 ± 9.5 years) were included in the statistical analysis. The variables analyzed to assess the effectiveness of the Elon IOL included high-contrast visual acuity, contrast sensitivity, the defocus curve, and visual symptoms. **Results**: Three months after implantation, the mean residual sphere was 0.00 ± 0.33 D, while the mean residual cylinder was −0.25 ± 0.41 D. Without correction, patients achieved monocular decimal visual acuity values of 0.94 ± 0.26 for distance, 0.79 ± 0.17 for intermediate, and 0.58 ± 0.15 for near vision. The mean uncorrected contrast sensitivity was 1.61 ± 0.15 log. The defocus curve showed visual acuity exceeding 0.80 decimal (0.10 logMAR) over a 2.00 D range and above 0.63 decimal (0.20 logMAR) over a 2.50 D range. The most frequently reported symptoms, with mild severity and bothersomeness, were glare, starbursts, halos, and focusing difficulties. **Conclusions**: Patients implanted with the Elon IOL achieved satisfactory visual quality at all distances, comparable to outcomes reported for other EDoF IOLs in the scientific literature.

## 1. Introduction

Over the last decade, the implantation of intraocular lenses (IOLs) with extended depth-of-focus (EDoF) designs became a solid alternative to multifocal IOLs with diffractive and refractive designs for correcting both refractive errors and presbyopia after cataract extraction [1]. The optical principle of EDoF IOLs involves creating a retinal image with a single extended focus, evenly distributing light energy across all visual distances [2]. Optically, this means that the spherical aberration induced by these lenses would be less dependent on pupil size, and visually, it would reduce overlapping retinal images [3,4].

Currently, there are dozens of EDoF IOLs on the market with a wide variety of optical designs, all sharing a continuous optical profile. It might be thought that the extended focus of EDoF IOLs optimizes patients’ intermediate vision, offering a more stable visual experience across all distances [5,6]. However, when their visual performance is compared with trifocal IOLs, which represent the other predominant multifocal IOLs, scientific evidence does not support a general improvement in visual quality or a reduction in visual symptoms with EDoF IOLs [7,8].

Therefore, it is essential to individually assess the visual performance of each available EDoF IOL to identify their advantages and limitations, ensuring their successful implantation in patients. In this regard, the aim of this study was to evaluate the visual quality and symptomatology of a non-diffractive EDoF IOL, the Elon 877PEY (Medicontur; Zsámbék, Hungary), three months after its implantation. To achieve this, visual acuity, contrast sensitivity, the defocus curve, and patient-reported symptoms were assessed. The difference between the Elon IOL and other EDoF lenses lies in its transition surfaces between the concentric refractive zones, which vary in curvature and allow for a smooth, continuous transition between zones. As a non-diffractive design, the central optic zone of this IOL is composed of concentric refractive zones that distribute light energy continuously along the optical axis.

Recently, Ferrando-Gil et al. [9] conducted a pilot study assessing visual quality and symptomatology in 10 patients implanted with the Elon IOL three months post-surgery. While their preliminary results provided an initial understanding of the lens’s performance, the limited sample size restricted the generalizability of their findings. In this context, the present study aims to expand and strengthen the existing evidence by analyzing a substantially larger sample (n = 28).

## 2. Materials and Methods

A cross-sectional case series study was conducted, with measurements taken three months after the implantation of the Elon IOL. The study adhered to current European regulations and the principles outlined in the Declaration of Helsinki. Approval of the study was obtained from the research ethics committee of Hospital Arruzafa (code: 0,1ELON; approval date: 24 March 2023). All procedures were performed at Hospital Arruzafa (Córdoba, Spain). Patients voluntarily participated after signing an informed consent form that provided comprehensive details about cataract surgery, IOL implantation, and the use of their data for scientific purposes.

A total of 28 patients (mean age: 64.5 ± 9.5 years) were included in the statistical analysis. All participants underwent bilateral implantation of the Elon IOL. Inclusion criteria were as follows: age between 45 and 90 years, presence or absence of any degree of cataract, preoperative astigmatism below 1.25 D, a clear desire to receive a multifocal IOL, and agreement to and understanding of the informed consent form. Exclusion criteria included a history of previous ocular surgery (including refractive surgery), ocular pathologies contraindicating multifocal IOL implantation, the use of systemic or ocular medications affecting vision or contraindicating surgery, and any intraoperative complications. Among the 28 included patients, 1 presented with bilateral high myopia (without pathological findings), 2 with bilateral cornea guttata, 1 with bilateral retinal drusen (without visual impairment), 1 with mild alterations of the retinal pigment epithelium (without visual impairment), and 1 with bilateral vitreous follicles.

Table 1 provides the specifications of the implanted Elon IOL, which features a non-diffractive EDoF optic design. The IOL was implanted via a standard phacoemulsification procedure, performed by the same experienced surgeons (A.C.-O., A.S.-V. and A.V.). After the crystalline lens was aspirated and irrigated, the lens was implanted using the manufacturer’s preloaded Bi-Flex POB-MA injector through a 2.2 mm corneal incision. The IOL power was calculated using the Barret TK formula, targeting a postoperative spherical equivalent of 0.00 D for both eyes.

High-contrast visual acuity was measured for distance (4 m), intermediate (67 cm), and near vision (40 cm) using the ETDRS original Series Chart 1 (Precision Vision; Woodstock, IL, USA), both corrected and uncorrected. Each optotype contained five letters per line of visual acuity, and measurements ceased when patients failed or could not read at least four letters on the same line [10].

Contrast sensitivity was evaluated with the Pelli–Robson test (Clement Clarke International; Harlow, UK), positioned 1 m from the patient. For this distance, the spatial frequency of the letters corresponds to 1 cycle/degree. The Pelli–Robson test includes 16 triplets of letters with a contrast reduction of 0.15 log between triplets. Patients were asked to read the letters, starting from the highest-contrast triplet, until they failed or could not read at least two letters in a triplet [11].

Binocular defocus curves were measured using the Multifocal Lens Analyzer app (Qvision; Almeria, Spain) for iPad (Apple Inc.; Cupertino, CA, USA). Visual acuity was assessed with the ETDRS test included in the app, covering defocus values from +1.00 D to −4.00 D following a semi-automated procedure [12].

Visual symptoms were assessed using the Quality of Vision (QoV) questionnaire, which evaluates the frequency, severity, and bothersome of symptoms such as glare, starburst, blurred vision, double vision, halos, hazy vision, distortion, vision fluctuation, focusing difficulties, and difficulty judging distances. The questionnaire rates frequency as never (0), occasionally (1), quite often (2), and very often (3); severity as not at all (0), mild (1), moderate (2), and severe (3); and bothersome as: not at all (0), a little (1), quite (2), and very (3) [13].

Statistical analysis was performed using the SPSS Statistics 29 software (IBM; Armonk, NY, USA) to present the descriptive outcomes of qualitative and quantitative variables. The variables analyzed to assess the effectiveness of the Elon IOL implantation included visual acuity, contrast sensitivity, the defocus curve, and visual symptoms.

## 3. Results

Of the 29 patients initially considered, 1 was excluded due to prior LASIK surgery. The final sample consisted of 28 Caucasian patients who underwent bilateral implantation of the Elon IOL. Table 2 provides the demographic and clinical characteristics of these patients, six of whom did not have any degree of cataract before IOL implantation.

**Figure 1:**
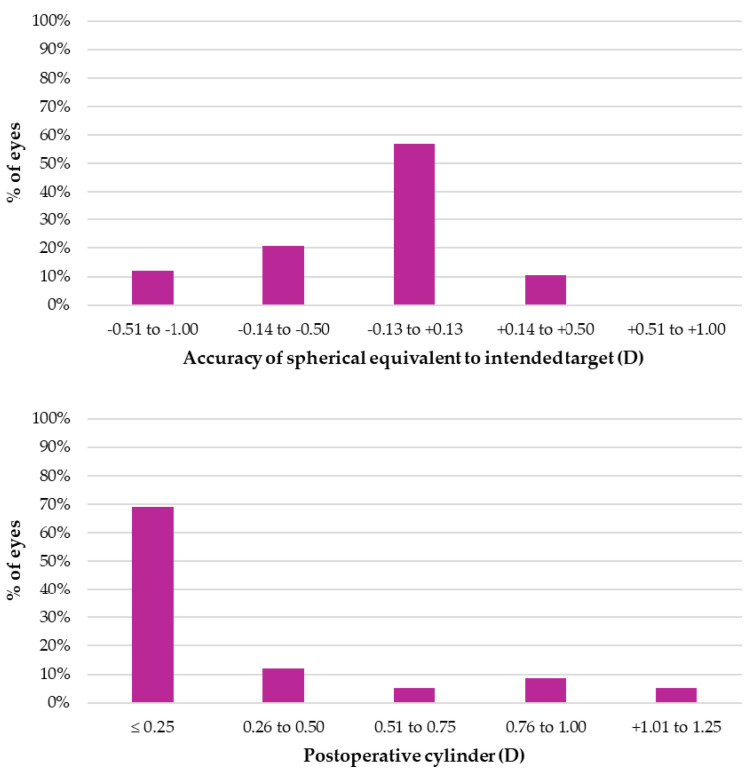
Refractive outcomes three months after surgery.

Figure 1 shows the refractive outcomes in terms of the spherical equivalent and cylinder three months after surgery. A total of 87.9% of eyes achieved a spherical equivalent within ±0.50 D, and 81.1% had a cylinder of ≤ 0.50 D. The mean residual sphere was 0.00 ± 0.33 D, while the mean residual cylinder was −0.25 ± 0.41 D.

Visual function outcomes, encompassing visual acuity and contrast sensitivity, are summarized in Table 3. Without correction, patients obtained monocular decimal visual acuity values of 0.94 ± 0.26 for distance, 0.79 ± 0.17 for intermediate, and 0.58 ± 0.15 for near vision. The mean uncorrected contrast sensitivity was 1.61 ± 0.15 log.

Notably, 86% of patients achieved a binocular visual acuity of 1.0 decimal or better for distance vision, 82% achieved 0.8 decimal or better for intermediate vision, and 82% achieved 0.6 decimal or better for near vision. Furthermore, none of the patients required near vision spectacles during the three-month follow-up period.

**Figure 2:**
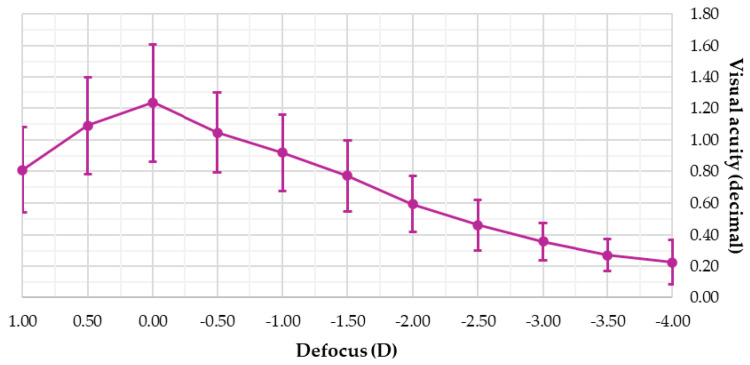
Defocus curve three months after surgery.

Figure 2 presents the defocus curve for the 28 patients three months after surgery. A maximum visual acuity of 1.24 ± 0.37 decimal was recorded without defocus (0.00 D). At a defocus of −1.50 D (67 cm), visual acuity was 0.77 ± 0.22 decimal, and at −2.50 D (40 cm), it was 0.46 ± 0.16 decimal. The defocus curve indicates visual acuity exceeding 0.80 decimal (0.10 logMAR) over a 2.00 D range (from +1.00 D to −1.00 D) and above 0.63 decimal (0.20 logMAR) over a 2.50 D range (from +1.00 D to −1.50 D).

Table 4 details the results of the visual symptoms reported by patients as evaluated using the QoV questionnaire. The most frequently reported symptoms with the highest severity and bothersomeness were glare, starburst, halos, and focusing difficulties. In terms of frequency, 59.3% of patients reported no or only occasional glare, 77.8% reported the same for starburst, 59.3% for halos, and 85.2% for focusing difficulties. Regarding severity, 63% of patients experienced no severity or only mild severity for glare, 77.8% for starburst, 70.4% for halos, and 88.9% for focusing difficulties. For bothersome symptoms, 85.2% of patients reported no bother or only slight bother for glare, 88.9% for starburst, 85.2% for halos, and 88.9% for focusing difficulties. On the other hand, double vision, distortion, and difficulty judging distance were reported as the least significant symptoms.

## 4. Discussion

This study evaluated the clinical efficacy of a new non-diffractive EDoF IOL, the Elon 877PEY, implanted in patients undergoing phacoemulsification. This IOL demonstrated proper visual quality for distance vision, which progressively decreased toward near distances in a manner that remained tolerable for patients. In this section, the results of each studied variable will be discussed in detail, comparing them with those obtained by other EDoF and trifocal IOLs.

In terms of uncorrected visual acuity, the Elon IOL provided decimal values close to 1.0 monocularly and higher than 1.0 binocularly. Compared to these values, intermediate and near visual acuities decreased by approximately 0.2 and 0.4 decimal units, respectively, compared to distance vision. Observing the defocus curve confirms this trend of decreasing visual quality with shorter visual distances, but it does so progressively, ensuring more stable vision for the patients. These findings may explain why patients reported minimal symptoms of double vision, related to possible overlapping retinal images, but experienced mild focusing difficulties. These uncorrected visual acuity results are consistent with those reported by Ferrando-Gil et al. [9] in a previous pilot study evaluating the efficacy of the Elon IOL in only 10 implanted patients.

When comparing the visual acuity results with those reported in other studies in the scientific literature, it was found that, for distance vision, the Elon IOL demonstrated visual acuity comparable to that of other IOLs, both EDoF and trifocal [14,15,16,17], and even slightly better than that reported by Cochener et al. [18]. For intermediate vision, greater variability was observed in this comparison, with the Elon IOL showing better visual performance than some EDoF and trifocal IOLs [14,18] but worse than others [16,17]. For near vision, the Elon IOL achieved visual acuity comparable to that of other EDoF IOLs [14,16], even improving by one decimal line compared to some designs [15,17,18], although it was slightly inferior to that of trifocal IOLs [14,15,16,17]. These referenced studies are among the most representative in the current scientific literature; however, our comparisons cannot be generalized to all EDoF or trifocal designs available on the market, emphasizing the need for individual assessments in each case.

The contrast sensitivity results measured with the Pelli–Robson test showed that patients implanted with the Elon IOL had uncorrected values similar to normal levels previously reported in individuals over 60 years of age [11,19]. These normal values were around 1.70 log, slightly better than those obtained by the patients in the current study (1.61 log). This would indicate that the loss of contrast sensitivity with the Elon IOL for spatial frequencies of 1 cpd is minimal. However, in the previous pilot study by Ferrando-Gil et al. [9], the Elon IOL appeared to reduce contrast sensitivity at high spatial frequencies (12 and 18 cpd), a limitation similarly observed with other currently available EDoF IOLs [20,21].

As previously mentioned, the defocus curve shows that visual acuity progressively decreases as the viewing distance shortens. The curve profile is typical of other EDoF IOLs, which usually exhibit a steeper slope compared to trifocal IOLs presenting a continuous curve. This suggests that EDoF IOLs may offer better visual acuity from far to intermediate distances, while trifocal IOLs would perform better in near vision [22]. In the case of the Elon IOL, the greatest loss of visual acuity, nearly two decimal lines, occurred when defocus changed from −1.50 D (67 cm) to −2.00 D (50 cm). This loss of visual acuity slightly contrasts with the profile of other EDoF IOLs, where the loss occurs between −2.00 D (50 cm) and −2.50 D (40 cm), providing a wider range of clear vision for intermediate to near distances [8,23]. Since the Elon IOL achieved visual acuities higher than 1.0 for positive defocus, one possibility to improve near visual acuity without compromising distance vision in future surgeries would be to shift the profile to the right, targeting a postoperative spherical equivalent of −0.25 D or −0.50 D. On the other hand, the defocus curve profile of the Elon IOL in the current sample was identical to that found in the pilot study conducted by Ferrando-Gil et al. [9], with the only significant difference being that distance visual acuity was approximately one line better in our study.

Regarding the symptomatology reported by the patients, the most frequent, severe, and bothersome visual symptoms were those inherent to the multifocal optics of EDoF IOLs (glare, halo, and starburst), as well as the expected focusing difficulties due to the reduced intermediate-to-near visual acuity inherent to these optical designs. The symptoms observed with the Elon IOL were consistent with previously published studies on other EDoF IOLs, which also found that glare, halo, and starburst were the most frequently reported symptoms [14,24]. Additionally, mild focusing difficulties could be attributed to the reduction in visual acuity observed at 50 cm.

During the three-month follow-up period of this study, patients did not require the use of near addition spectacles, and none of them experienced intolerance to the Elon IOL. However, it should be noted that, according to the findings reported by Ferrando-Gil et al. [9] in their pilot study, although patients exhibit acceptable levels of visual symptoms, they may experience difficulties in certain daily activities, particularly driving at night or in rainy conditions. In this regard, the current study lacks additional questionnaires to assess the visual impact of the Elon IOL on the daily lives of implanted patients, which would provide further insight into a larger sample.

Finally, it is important to highlight some of the main limitations of this study. Firstly, this was a cross-sectional case series study in which the outcomes of the Elon IOL were not directly compared to those of any other EDoF or trifocal IOL. Although a case series does not represent the highest level of scientific evidence in clinical research, this study provides an initial contribution that allows for direct comparison with findings from the existing scientific literature. Furthermore, the sample of patients who received the lens was heterogeneous in terms of age, including individuals in their early 50s undergoing phacoemulsification of a clear crystalline lens as well as elderly individuals over 80 years older who presented with advanced stages of cataracts. This variability directly influenced the differences observed in both visual acuity and contrast sensitivity, parameters that are inevitably affected by age. Additionally, it is worth noting that the results of this study are based on a follow-up period of only three months, leaving uncertainty about the long-term outcomes in patients whose visual quality could potentially deteriorate, possibly leading to a dependence on spectacles, especially in those daily activities where visual quality may be compromised, such as the previously mentioned case of driving at night or in rainy conditions.

## 5. Conclusions

Patients implanted with the Elon IOL achieved adequate visual quality at all distances, comparable to the outcomes reported for other EDoF IOLs in the scientific literature. However, it is important to note a significant reduction in near visual acuity at 50 cm, which was well tolerated by patients during the first three months following implantation. This limitation could potentially be mitigated in future procedures by targeting a slightly myopic postoperative spherical equivalent, without compromising distance visual acuity.

## Figures and Tables

**Table 1:** Characteristics of the EDoF intraocular lens used in the study.

**Technical name**	Elon 877PEY
**Manufacturer**	Medicontur (Zsámbék, Hungary)
**Material**	Hydrophobic acrylic
**Overall diameter (mm)**	13.00
**Optic diameter (mm)**	6.00
**Optic design**	Biconvex aspheric
**Haptic design**	Posterior vaulting fenestrated C-loop with 0° angulation
**Refractive index**	1.47
**Abbe number**	58
**A constant (SRK/T)**	118.9
**Standard powers (D)**	+8.0 D, +30.0 D (0.5 D steps)
**Extreme powers (D)**	+31.0 D, +35.0 D (1.0 D steps)
**Filtration**	UV, blue light

Data are available from the manufacturer.

**Table 2:** Demographic and preoperative refractive characteristics of patients.

Variable	Mean ± SD	Range
Participants (n)	28	-
Eyes analyzed (n)	56	-
Gender (male, %)	52%	-
Working status (active, %)	62%	-
Age (years)	64.5 ± 9.5	[47, 86]
UDVA (decimal)	0.20 ± 0.26	[0.00, 0.90]
CDVA (decimal)	0.84 ± 0.23	[0.50, 1.00]
Sphere (D)	1.09 ± 3.17	[−11.25, +7.75]
Cylinder (D)	−0.62 ± 0.45	[−1.25, 0.00]

UDVA: uncorrected distance visual acuity; CDVA: corrected distance visual acuity.

**Table 3:** Visual acuity and contrast sensitivity three months after surgery.

Variable	Mean ± SD (Decimal)	Range (Decimal)
UDVA monocular	0.94 ± 0.26	[0.40, 1.66]
UDVA binocular	1.11 ± 0.33	[0.80, 1.66]
CDVA monocular	1.05 ± 0.20	[0.63, 1.66]
CDVA binocular	1.19 ± 0.22	[0.90, 1.66]
UIVA monocular	0.79 ± 0.17	[0.30, 1.00]
UIVA binocular	0.90 ± 0.16	[0.50, 1.20]
CIVA monocular	0.82 ± 0.18	[0.40, 1.00]
CIVA binocular	0.88 ± 0.22	[0.60, 1.20]
UNVA monocular	0.60 ± 0.17	[0.25, 1.00]
UNVA binocular	0.69 ± 0.14	[0.50, 1.00]
CNVA monocular	0.58 ± 0.15	[0.30, 0.90]
CNVA binocular	0.65 ± 0.17	[0.30, 0.90]
CS monocular (log)	1.61 ± 0.15	[1.20, 1.80]
CS binocular (log)	1.77 ± 0.14	[1.50, 1.95]

UDVA: uncorrected distance visual acuity; CDVA: corrected distance visual acuity; UIVA: uncorrected intermediate visual acuity; CIVA: corrected intermediate visual acuity; UNVA: uncorrected near visual acuity; CNVA: corrected near visual acuity; CS: uncorrected contrast sensitivity.

**Table 4:** Visual symptoms assessed by the QoV questionnaire three months after surgery.

Symptom	Mean ± SD (Score)		
	Frequency	Severity	Bothersome
Glare	1.1 ± 1.0	1.1 ± 1.0	0.8 ± 0.9
Starburst	1.0 ± 1.0	0.9 ± 0.9	0.7 ± 0.9
Blurred vision	0.6 ± 0.8	0.6 ± 0.7	0.4 ± 0.6
Double vision	0.1 ± 0.3	0.1 ± 0.3	0.1 ± 0.3
Halos	1.2 ± 1.2	1.0 ± 1.0	0.9 ± 0.9
Hazy vision	0.6 ± 0.7	0.6 ± 0.8	0.5 ± 0.6
Distortion	0.2 ± 0.6	0.2 ± 0.6	0.2 ± 0.6
Vision fluctuation	0.4 ± 0.6	0.3 ± 0.5	0.2 ± 0.4
Focusing difficulties	0.9 ± 0.9	0.6 ± 0.8	0.6 ± 0.8
Difficult judging distance	0.1 ± 0.4	0.1 ± 0.4	0.1 ± 0.4

## Data Availability

The raw data supporting the conclusions of this article will be made available by the authors on reasonable request.

## References

[1] Schnider C., Yuen L., Rampat R., Zhu D., Dhallu S., Trinh T., Gurnani B., Abdelmaksoud A., Bhogal-Bhamra G., Wolffsohn J.S. (2024). BCLA CLEAR presbyopia: Management with intraocular lenses. Contact Lens Anterior Eye.

[2] Kanclerz P., Toto F., Grzybowski A., Alio J.L. (2020). Extended Depth-of-Field Intraocular Lenses: An Update. Asia Pac. J. Ophthalmol..

[3] Azor J.A., Vega F., Armengol J., Millan M.S. (2022). Optical Assessment and Expected Visual Quality of Four Extended Range of Vision Intraocular Lenses. J. Refract. Surg..

[4] Fernández-Núñez S., Pérez-Sanz L., Gómez-Pedrero J.A., García-Montero M., Albarrán-Diego C., Garzón N. (2024). Optical quality in vitro and in vivo of an extended depth-of-focus intraocular lens with isofocal design. Graefes Arch. Clin. Exp. Ophthalmol..

[5] Rampat R., Gatinel D. (2021). Multifocal and Extended Depth-of-Focus Intraocular Lenses in 2020. Ophthalmology.

[6] Megiddo-Barnir E., Alió J.L. (2023). Latest Development in Extended Depth-of-Focus Intraocular Lenses: An Update. Asia Pac. J. Ophthalmol..

[7] Karam M., Alkhowaiter N., Alkhabbaz A., Aldubaikhi A., Alsaif A., Shareef E., Alazaz R., Alotaibi A., Koaik M., Jabbour S. (2023). Extended Depth of Focus Versus Trifocal for Intraocular Lens Implantation: An Updated Systematic Review and Meta-Analysis. Am. J. Ophthalmol..

[8] Tavassoli S., Ziaei H., Yadegarfar M.E., Gokul A., Kernohan A., Evans J.R., Ziaei M. (2024). Trifocal versus extended depth of focus (EDOF) intraocular lenses after cataract extraction. Cochrane Database Syst. Rev..

[9] Ferrando Gil J., Churruca Irazola A., Reparaz I., Lauzirika G., Martínez-Soroa I., Mendicute J. (2024). Visual, Refractive, Functional, and Patient Satisfaction Outcomes After Implantation of a New Extended Depth-of-Focus Intraocular Lens. Clin. Ophthalmol..

[10] Kniestedt C., Stamper R.L. (2003). Visual acuity and its measurement. Ophthalmol. Clin. N. Am..

[11] Mäntyjärvi M., Laitinen T. (2001). Normal values for the Pelli-Robson contrast sensitivity test. J. Cataract. Refract. Surg..

[12] Rodríguez-Vallejo M., Burguera N., Rocha-de-Lossada C., Aramberri J., Fernández J. (2023). Refraction and defocus curves in eyes with monofocal and multifocal intraocular lenses. J. Optom..

[13] McAlinden C., Pesudovs K., Moore J.E. (2010). The Development of an Instrument to Measure Quality of Vision: The Quality of Vision (QoV) Questionnaire. Investig. Ophthalmol. Vis. Sci..

[14] Monaco G., Gari M., Di Censo F., Poscia A., Ruggi G., Scialdone A. (2017). Visual performance after bilateral implantation of 2 new presbyopia-correcting intraocular lenses: Trifocal versus extended range of vision. J. Cataract. Refract. Surg..

[15] Gil M.A., Varón C., Cardona G., Buil J.A. (2020). Visual acuity and defocus curves with six multifocal intraocular lenses. Int. Ophthalmol..

[16] Webers V.S.C., Bauer N.J.C., Saelens I.E.Y., Creten O.J.M., Berendschot T., van den Biggelaar F., Nuijts R. (2020). Comparison of the intermediate distance of a trifocal IOL with an extended depth-of-focus IOL: Results of a prospective randomized trial. J. Cataract. Refract. Surg..

[17] Scheepers M.A., Bunce C.B., Michaelides M., Hall B. (2023). Clinical outcomes of a trifocal compared with an extended depth of focus IOL following bilateral cataract surgery. Can. J. Ophthalmol..

[18] Cochener B., Boutillier G., Lamard M., Auberger-Zagnoli C. (2018). A Comparative Evaluation of a New Generation of Diffractive Trifocal and Extended Depth of Focus Intraocular Lenses. J. Refract. Surg..

[19] Hirvelä H., Koskela P., Laatikainen L. (1995). Visual acuity and contrast sensitivity in the elderly. Acta Ophthalmol. Scand..

[20] Ang R.E., Picache G.C.S., Rivera M.C.R., Lopez L.R.L., Cruz E.M. (2020). A Comparative Evaluation of Visual, Refractive, and Patient-Reported Outcomes of Three Extended Depth of Focus (EDOF) Intraocular Lenses. Clin. Ophthalmol..

[21] Gundersen K.G., Potvin R. (2020). Comparing Visual Acuity, Low Contrast Acuity and Contrast Sensitivity After Trifocal Toric and Extended Depth of Focus Toric Intraocular Lens Implantation. Clin. Ophthalmol..

[22] Fernández J., Ribeiro F., Rocha-de-Lossada C., Rodríguez-Vallejo M. (2024). Functional Classification of Intraocular Lenses Based on Defocus Curves: A Scoping Review and Cluster Analysis. J. Refract. Surg..

[23] Liu J., Dong Y., Wang Y. (2019). Efficacy and safety of extended depth of focus intraocular lenses in cataract surgery: A systematic review and meta-analysis. BMC Ophthalmol..

[24] Corbett D., Black D., Roberts T.V., Cronin B., Gunn D., Bala C., Versace P., Tsai L., Papadatou E., Alarcon A. (2024). Quality of vision clinical outcomes for a new fully-refractive extended depth of focus Intraocular Lens. Eye.

